# Supplementary material for: COVID-19, individual wellbeing and multi-dimensional poverty in the state of South Australia
Source: PLoS One. 2021 Jun 10;16(6):e0252898. doi: 10.1371/journal.pone.0252898 (PMC8191965; doi:10.1371/journal.pone.0252898)
Supplement: S1 File — (PDF) [file pone.0252898.s001.pdf]

Introduction and Consent

Flinders University wants to know about your experiences during COVID-19.

This survey will help us discover how COVID-19 has affected people living in different areas of South Australia. It asks questions about your activities, your work, and your living situation. By asking questions about more than just health, we can get a better idea of how well people are really doing. We hope this information can help improve the lives of South Australians both during and after COVID-19.

The survey will take about 10 minutes and participation is entirely voluntary. You may choose to not complete it or not submit your answers at any time. As the survey is anonymous, no personally identifying information will be collected or retained. For additional information please see the Survey information consent document.

- ◯ I have read and understood the Survey Information Consent document for this project and confirm that I am over the age of 18.

Base Questions

Which Council area do you currently live in?

- ◯ Mitcham
- ◯ Salisbury
- ◯ Goyder Regional
- ◯ Other

What suburb do you currently live in?

What is your gender?

- Male
- Female
- Other

What is your age?

- 15-24
- 25-29
- 30-49
- 50-64
- 65 and above

Do you live with your family or others in your household?

- Yes, I live with family
- Yes, I live with others
- No, I live by myself

If you live by yourself, are you thinking of moving into your family household or a shared household due to financial or emotional factors related to COVID-19?

- Yes
- No

If you are currently living with others in a shared household, did you move there due to COVID-19?

- Yes
- No

If you are currently living in a family household, did you move there due to COVID-19?

- Yes
- No

Are you an Australian Citizen or a permanent resident of Australia?

- Yes
- No

At the outbreak of COVID-19, what was your occupation?

- Student
- Own business
- Government employee
- Teacher
- University/TAFE academic
- Construction
- Medical/aged care
- Hospitality
- Other
- Not applicable

What is your current weekly income?

- No income
- Less that $200
- Between $200-$400
- Between $401-$600
- More than $600

Overall Wellbeing

On a scale of 1-8, before the period of self-isolation/lockdown, how positive or hopeful were you with your life?

- 1 (not positive/hopeful)
- 2
- 3
- 4
- 5
- 6
- 7
- 8 (very positive/hopeful)

On a scale of 1-8, during and after the period of self-isolation/lockdown, how positive and hopeful were you with your life?

- 1 (not positive/hopeful)
- 2
- 3
- 4
- 5
- 6
- 7
- 8 (very positive/hopeful)

Psychological Wellbeing

Since March 2020, due to the widespread news coverage or the restrictions related to COVID-19, have you been depressed or anxious?

- Yes
- No

Since March 2020, due to the widespread news overage or the restrictions related to COVID-19, have you experienced problems with sleeping?

- Yes
- No

Since March 2020, due to widespread news coverage or the restrictions related to COVID-19, have you experienced any problem with concentrating?

- Yes
- No

Since March 2020, due to COVID-19, have you been drinking more alcohol and /or smoking more tobacco than usual?

- Yes
- No

Since March 2020, due to COVID-19, have you experienced an increase in interpersonal conflict in your household?

- Yes
- No

Overall, what kind of impact do you feel that the COVID-19 pandemic has on your mental health?

- Positive
- Negative
- No impact

Physical Health Wellbeing

Since March 2020, have you tended to maintain familiar routines in daily life, e.g. regular mealtimes, bedtime?

- Yes
- No

Since March 2020, have you been consciously doing physical exercise?

- Yes
- No

Since March 2020, have you consciously tried to boost your immune system by taking vitamins or/and by improving your daily food-based nutrition?

- Yes
- No

Since March 2020, did you experience any interruption to ongoing health related treatment due to government restrictions on movements and gatherings?

- Yes
- No

Did you have to cut down your health-related expenditure due to financial pressure (such as loss of income due to COVID-19) since March 2020?

- Yes
- No

Overall, do you feel that the COVID-19 pandemic has had a negative impact on your physical health?

- Yes
- No

Living Standards

How has your income been affected due to COVID-19?

- Went up
- Went down
- Remained the same

How has your financial savings been impacted due to COVID-19?

- Went up
- Went down
- Remained the same

Did you have to cut down the consumption of your usual food items due to financial pressure (such as loss of income due to COVID-19) since March 2020?

- Yes
- No

Since March 2020, have you been unable to pay your accommodation rent or mortgage in full?

- Yes
- No

Did you receive any financial support from the Government (JobSeeker or JobKeeper allowance)?

- Yes
- No, although I am eligible
- No, I am not eligible

Due to COVID-19, how do you feel about your job security right now?

- More secure
- Less secure
- No change
- Don't know
- Not applicable

Overall, do you feel that COVID-19 has had a negative impact on your living standard?

- Yes
- No

Family/Community Vitality

During COVID-19, have you been able to draw on the support of friends and family to help you deal with difficult situations and share with them your worries and concerns?

- Yes
- No
- Did not require it

During COVID-19, how often have you kept in regular contact with loved ones not in your household via telephone, e-mail, social media, or other distance communication mechanism?

- Everyday
- At least once every week
- A few times a month
- A few times during the entire period
- Not at all

During COVID-19, have you checked on neighbours or people in your community who may need some extra assistance, via telephone, email, social media, or other

distance communication mechanism?

- Yes
- No

During COVID-19, how often you have you stayed connected with your social network via telephone, e-mail, social media or video conference?

- Every day
- At least once every week
- A few times a month
- A few times during the entire period
- Not at all

During COVID-19, have you provided assistance/reached out to people from a different cultural background who may need some extra assistance (this could be through donations, volunteering, for example?)

- Yes
- No
- Did not get an opportunity to do so

Do you think everyone who is currently in Australia (irrespective of their citizenship status, ethnicity, etc.), should be given equal opportunity to access Government assistance to address the financial impacts of COVID-19?

- Yes
- No
- Not sure

Education

During COVID-19, was/is your school/TAFE/university temporarily closed for classes?

- Yes

- ◯ No
- ◯ Not applicable

How do you rate the impact of the COVID-19 pandemic on your education?

- ◯ Major
- ◯ Moderate
- ◯ Minor
- ◯ Not applicable

How do you rate the impact of the COVID-19 pandemic on your children's education?

- ◯ Major
- ◯ Moderate
- ◯ Minor
- ◯ Not applicable

If you are at school/university/TAFE, then did you or are you home-schooling or currently doing the courses online due to the COVID-19 situation?

- ◯ Yes
- ◯ No
- ◯ Not applicable

If there was/is home-schooling in your household due to COVID-19, who was/is helping with the lessons (check all that apply)?

- ☐ Mother
- ☐ Father
- ☐ Sibling
- ☐ Other
- ☐ Not applicable

Governance

How do you rate your attitude towards Government policies on managing the COVID-19 pandemic (i.e.: social distancing, mask wearing, self-isolation, etc.)?

- Very accepting
- Moderately accepting
- Resentful

How do you rate the Government's policies on COVID-19 restrictions? (check all that apply)

- Restrictions were appropriate
- Restrictions were withdrawn too early
- Restrictions should not have been withdrawn
- Restrictions should be stronger
- Restrictions are too strong
- There should be no restrictions

How do you rate your level of trust in the Government's information about COVID 19?

- 1 (very low)
- 2
- 3
- 4
- 5 (very high)

How do you rate the performance of your local Council in dealing with the pandemic situation?

- 1 (very low)
- 2
- 3
- 4

- 5 (very high)

How do you rate the overall performance of the South Australian Government in dealing with the pandemic situation?

- 1 (very low)
- 2
- 3
- 4
- 5 (very high)

How do you rate the overall performance of the Federal Government in dealing with the pandemic situation?

- 1 (very low)
- 2
- 3
- 4
- 5 (very high)

Do you think your overall situation would have been worse without any support from the Government?

- Yes
- No
- Not sure

Ecological Diversity and Resilience

During COVID-19, did you spend more time than usual in your personal/household garden or outdoor area?

- Yes

- No
- Do not have a garden or outdoor area

During COVID-19, did you make more use than usual of public parks or outdoor areas near where you live?

- Yes
- No
- There are no public parks/outdoor areas near me

During COVID-19, how much has the use of outdoor spaces contributed to your wellbeing?

- A lot
- Some
- Not at all

During COVID-19, how often have you engaged in outdoor activities such as walking, hiking, cycling, beach swimming/surfing, etc.?

- Frequently
- Occasionally
- Not at all

Do you feel your health or wellbeing has improved due to reduction in emissions during COVID-19?

- Yes
- No
- Emissions have not changed where I am

Do you think people should voluntarily reduce the use of cars to help reduce emissions after the COVID-19 restrictions end?

- Yes
- No

Did your view on people voluntary reducing the use of cars change due to COVID-19?

- Yes
- No

Have you found that your pet animal(s) have been a benefit to your wellbeing during COVID-19?

- Very much
- Somewhat
- Not at all
- Do not have any pet animal

Are you concerned about the amount of individual household waste produced during COVID-19?

- Yes
- No

Exit

Would you like to review or change your answers before submitting?

- Yes, return me to the beginning of my survey
- No, submit now

Powered by Qualtrics
