# Supplementary material for: COVID-19, individual wellbeing and multi-dimensional poverty in the state of South Australia
Source: PLoS One. 2021 Jun 10;16(6):e0252898. doi: 10.1371/journal.pone.0252898 (PMC8191965; doi:10.1371/journal.pone.0252898)
Supplement: Attachment — Submitted filename: Response to Reviewers.docx [file pone.0252898.s002.docx]

**PONE-D-21-01771**

**COVID-19, individual wellbeing and multi-dimensional poverty in the state of South Australia**

**PLOS ONE**

**Response to Reviewers**

07/05/2021

Dear Sir/Madam,

As requested in your email of 28 April 2021, below please find a response to each of the points raised by the academic editor and reviewer of the manuscript indicated above.

**Academic Editor Comments**

1. Please ensure that your manuscript meets PLOS ONE's style requirements, including those for file naming.

*Authors’ Response: Manuscript and associated files have been updated accordingly.*

2. Please review your reference list to ensure that it is complete and correct. If you have cited papers that have been retracted, please include the rationale for doing so in the manuscript text, or remove these references and replace them with relevant current references. Any changes to the reference list should be mentioned in the rebuttal letter that accompanies your revised manuscript. If you need to cite a retracted article, indicate the article’s retracted status in the References list and also include a citation and full reference for the retraction notice.

*Authors’ Response: The reference list is complete, correct, and has been updated in the final Manuscript to reflect the required referencing and citation format. Please note these formatting changes are not reflected in the Revised Manuscript with Track Changes so as to reduce unnecessary confusion with the content changes in response to the Reviewers comments.*

3. Please include additional information regarding the survey or questionnaire used in the study and ensure that you have provided sufficient details that others could replicate the analyses.

*Authors’ Response: The questionnaire associated with this study has been provided as S1 File.*

4. We note that you have indicated that data from this study are available upon request. PLOS only allows data to be available upon request if there are legal or ethical restrictions on sharing data publicly. For more information on unacceptable data access restrictions, please see <http://journals.plos.org/plosone/s/data-availability#loc-unacceptable-data-access-restrictions>.

In your revised cover letter, please address the following prompts:

a) If there are ethical or legal restrictions on sharing a de-identified data set, please explain them in detail (e.g., data contain potentially sensitive information, data are owned by a third-party organization, etc.) and who has imposed them (e.g., an ethics committee). Please also provide contact information for a data access committee, ethics committee, or other institutional body to which data requests may be sent.

b) If there are no restrictions, please upload the minimal anonymized data set necessary to replicate your study findings as either Supporting Information files or to a stable, public repository and provide us with the relevant URLs, DOIs, or accession numbers. For a list of acceptable repositories, please see <http://journals.plos.org/plosone/s/data-availability#loc-recommended-repositories>*.*

*Authors’ Response: We have been advised by Flinders Research Ethics and Compliance that as the participants did not consent to sharing the data with other researchers/organisations, we are unable to provide it here, even in a de-identified form. Flinders Research Ethics and Compliance can be contacted at* [*human.researchethics@flinders.edu.au*](mailto:human.researchethics@flinders.edu.au)*.*

5. Please upload a copy of Figure 5, to which you refer in your text on page 9. If the figure is no longer to be included as part of the submission please remove all reference to it within the text.

*Authors’ Response: There is no Figure 5 associated with this manuscript. All references to such Figure have been modified or removed.*

**Additional Editor Comments:**

This manuscript is important and interesting. Please revise your manuscript according to the reviewer concerns.

[Note: HTML markup is below. Please do not edit.]

Reviewers' comments:

Reviewer's Responses to Questions

**Comments to the Author**

1. Is the manuscript technically sound, and do the data support the conclusions?

The manuscript must describe a technically sound piece of scientific research with data that supports the conclusions. Experiments must have been conducted rigorously, with appropriate controls, replication, and sample sizes. The conclusions must be drawn appropriately based on the data presented.

Reviewer #1: Yes

*Authors’ Response: None required.*

2. Has the statistical analysis been performed appropriately and rigorously?

Reviewer #1: Yes

*Authors’ Response: None required.*

3. Have the authors made all data underlying the findings in their manuscript fully available?

The [PLOS Data policy](http://www.plosone.org/static/policies.action#sharing) requires authors to make all data underlying the findings described in their manuscript fully available without restriction, with rare exception (please refer to the Data Availability Statement in the manuscript PDF file). The data should be provided as part of the manuscript or its supporting information, or deposited to a public repository. For example, in addition to summary statistics, the data points behind means, medians and variance measures should be available. If there are restrictions on publicly sharing data—e.g. participant privacy or use of data from a third party—those must be specified.

Reviewer #1: Yes

*Authors’ Comment: We have been advised by Flinders Research Ethics and Compliance that as the participants did not consent to sharing the data with other researchers/organisations, we are unable to provide it here, even in a de-identified form. Flinders Research Ethics and Compliance can be contacted at* [*human.researchethics@flinders.edu.au*](mailto:human.researchethics@flinders.edu.au)*.*

4. Is the manuscript presented in an intelligible fashion and written in standard English?

PLOS ONE does not copyedit accepted manuscripts, so the language in submitted articles must be clear, correct, and unambiguous. Any typographical or grammatical errors should be corrected at revision, so please note any specific errors here.

Reviewer #1: Yes

*Authors’ Response: None required.*

5. Review Comments to the Author

Please use the space provided to explain your answers to the questions above. You may also include additional comments for the author, including concerns about dual publication, research ethics, or publication ethics. (Please upload your review as an attachment if it exceeds 20,000 characters)

Reviewer #1: This is an important piece of research which throws light on how the situations arising out of COVID 19 affects individual well-being in a comprehensive way in South Australia. The research shows how the pandemic has cast multi-dimensional impacts on individuals’ well-being along with significant drivers of well-being. The paper suggests that while the majority of people in South Australia survive the ill-effect of the pandemic, about a third of the population is found to have been found to be “multi-dimensionally” affected with more than 3 dimensions of out of six being adversely affected simultaneously. This stresses upon the fact that the current pandemic is not only a health-crisis, rather more a social crisis. The paper goes on to argue that impacts of the pandemic have distinct gradients in terms of economic status, age and attitude towards life. The survey results reveal that the impacts have been disproportionately extreme in case of the young, poor and those with a weak attitude to life. Since, these are crucial survey findings, it will be better if the results of “formal statistical tests” so that these observed tendencies can be generalised.
The paper adopts a multi-dimensional framework in analysis with Alkire-Foster method as the guiding basis. The analysis is based double-cut-offs applying two-third rule and identification of the “multi-dimensionally” experiencing ill-being has been done in case of individuals who suffer in at-least 4 out of 6 dimensions together. The paper provides that 167 out of 579 are such individuals (i.e. 29 percent). This is the head-count. It will also be interesting to examine the “intensity” of ill-being – in the line of Alkire-Foster method – which will indicate the average number of dimensions that are being suffered by these 167 individuals. Table 4, similarly, gives the ‘censored percentages’. The paper may compare this by taking note of the “uncensored percentages” to see how different dimensions play out in the population as a whole.
I was just wondering whether individuals can be identified who have suffered from COVID 19 in the database. If we can have data on whether the individuals have suffered from COVID 19, or whether any of their family/relatives suffered from COVID 19 the results can further be disaggregated to see how they vary across these categories.
In summary, the paper is a valuable contribution and is recommended for publication with the above minor observations.

*Authors’ Response:*

*The results of the ‘formal statistical tests’, including income, attitude toward life, and age, can be found in Table 3: Socio-economic determinants of wellbeing – results of binomial logistic regression.*

*In response to the reviewer’s comment regarding ‘intensity’, the manuscript has now been modified to include additional discussion of this aspect of the research (pg. 15). Similarly, Table 4 now includes a column showing the ‘uncensored percentages’ for comparison.*

*With regard to identifying individuals who have suffered from COVID-19, this is not possible given the design of the survey, which did not ask participants to disclose this information.*

6. PLOS authors have the option to publish the peer review history of their article ([what does this mean?](https://journals.plos.org/plosone/s/editorial-and-peer-review-process#loc-peer-review-history)). If published, this will include your full peer review and any attached files.


If you choose “no”, your identity will remain anonymous but your review may still be made public.


**Do you want your identity to be public for this peer review?** For information about this choice, including consent withdrawal, please see our [Privacy Policy](https://www.plos.org/privacy-policy).

Reviewer #1: **Yes:** Joydeep Baruah

*Authors’ Response: None required.*

Should you need additional information or further clarification please do not hesitate to contact me.

Thank you for your time and interest in publishing this manuscript.

Yours sincerely

**Udoy Saikia**

[**udoy.saikia@flinders.edu.au**](mailto:udoy.saikia@flinders.edu.au)

**Corresponding author**
